# Genome sequencing and comparative genomics of honey bee microsporidia, *Nosema apis* reveal novel insights into host-parasite interactions

**DOI:** 10.1186/1471-2164-14-451

**Published:** 2013-07-05

**Authors:** Yan ping Chen, Jeffery S Pettis, Yan Zhao, Xinyue Liu, Luke J Tallon, Lisa D Sadzewicz, Renhua Li, Huoqing Zheng, Shaokang Huang, Xuan Zhang, Michele C Hamilton, Stephen F Pernal, Andony P Melathopoulos, Xianghe Yan, Jay D Evans

**Affiliations:** 1 USDA-ARS Bee Research Laboratory, Building 476 BARC-East, Beltsville, MD 20705, USA; 2 USDA-ARS Molecular Plant Pathology Laboratory, Beltsville, MD 20705, USA; 3 The Institute for Genome Sciences, University of Maryland School of Medicine, Baltimore, MD 21202, USA; 4 Windber Research Institute, 620 7th Street, Windber, PA 15963, USA; 5 College of Animal Sciences, Zhejiang University, Hangzhou, Zhejiang 310058, P.R. China; 6 College of Bee Science, Fujian Agriculture and Forestry University, Fuzhou, Fujian 350002, P.R. China; 7 Eastern Bee Research Institute of Yunnan Agriculture University, Kunming, Yunnan 650201, P.R. China; 8 Agriculture & Agri-Food Canada, Beaverlodge Research Farm, Beaverlodge, AB, Canada; 9 The Molecular Characterization of Foodborne Pathogens Unit, Eastern Regional Research Center (ERRC), Wyndmoor, PA 19038, USA

**Keywords:** Microsporidia, Nosema, Honey bees, Genome, Comparative genomics

## Abstract

**Background:**

The microsporidia parasite *Nosema* contributes to the steep global decline of honey bees that are critical pollinators of food crops. There are two species of *Nosema* that have been found to infect honey bees, *Nosema apis* and *N. ceranae*. Genome sequencing of *N. apis* and comparative genome analysis with *N. ceranae*, a fully sequenced microsporidia species, reveal novel insights into host-parasite interactions underlying the parasite infections.

**Results:**

We applied the whole-genome shotgun sequencing approach to sequence and assemble the genome of *N. apis* which has an estimated size of 8.5 Mbp. We predicted 2,771 protein- coding genes and predicted the function of each putative protein using the Gene Ontology. The comparative genomic analysis led to identification of 1,356 orthologs that are conserved between the two *Nosema s*pecies and genes that are unique characteristics of the individual species, thereby providing a list of virulence factors and new genetic tools for studying host-parasite interactions. We also identified a highly abundant motif in the upstream promoter regions of *N. apis* genes. This motif is also conserved in *N. ceranae* and other microsporidia species and likely plays a role in gene regulation across the microsporidia.

**Conclusions:**

The availability of the *N. apis* genome sequence is a significant addition to the rapidly expanding body of microsprodian genomic data which has been improving our understanding of eukaryotic genome diversity and evolution in a broad sense. The predicted virulent genes and transcriptional regulatory elements are potential targets for innovative therapeutics to break down the life cycle of the parasite.

## Background

Microsporidia are obligate intracellular parasites that comprise over 1,200 described species belonging to 143 genera and infect members of almost all animal phyla [1-3]. Until recently, microsporidia were regarded as primitive amitochondriate protozoa, because they do not possess typical eukaryotic organelles such as mitochondria, peroxisomes, and classical stacked Golgi apparati. In addition, ribosomal RNAs of microsporidia present some prokaryote-like features by harboring 16S and 23S rRNA genes and lacking the 5.8S rRNA in their large ribosomal subunit that is characteristic of eukaryotes [4]. However, this putatively ancient origin of microsporidia has been contested and redefined. Recent data has shown the presence of a mitosome, a mitochondrion-derived organelle [5], and genes encoding proteins of mitochondrial origin such as heat shock protein 70 in microsporidia [6,7]. This suggests that microsporidia once had mitochondria and lost them through the course of evolution. Based on the aforementioned factors, in conjunction with their possession of characteristic traits of closed mitosis and spores that contain chitin and trehalose, as fungi do [8], microsporidia have been classified as the earliest diverging clade of sequenced fungi [9] that underwent substantial genetic changes during adaptive evolution.

*Nosema apis* and *N. ceranae* (Family: Nosematidae, Genus: Nosema) are two microsporidia species that infect honey bees. Like other microsporidia, the infective form of the *Nosema* is the resistant spore, which is surrounded by an outer layer consisting of an electron-dense glycoprotein exospore and an electron-lucent chitinous endospore layer separated from the cell by a thin plasma membrane. The spore possesses a polar tubule which is considered to be a specialized product of the Golgi apparatus and the posterior vacuole at the posterior pole of the spore has been postulated to function as a peroxisome [10-12]. In the midgut of the host, the spore germinates and extrudes its polar tubule and injects the infective sporoplasm into the host intestinal epithelium. Inside the host cell cytoplasm, the sporoplasm undergoes divisions by binary fission (merogony) or multiple fission (schizogony) and spore production by formation of a thick wall around the spore (sporogony). Repeated multiplication results in the host cell becoming completely filled with spores and eventually rupturing to release the spores into surroundings cells [13] and to spread to other tissues [14,15]. Mature spores excreted in feces can also infect other hosts, providing new sources of the infection in bee colonies [16]. Parasitism by *Nosema* impairs the host’s metabolism and reproduction, and causes a destructive disease known as nosemosis, a digestive disorder that shortens honey bee lifespan, retards colony development, decimates bee populations, and causes colony queen supersedure [17-22]. *N. ceranae* as a destructive intracellular parasite not only directly causes a serious disease in honey bees but also has the potential to compromise the physical and immunological barriers of honey bees toward disease, leaving bees more susceptible to other pathogens and senescence [23-25]. New evidence shows that interactions between *N. ceranae* and neuro-active pesticides, neonicotinoids which have been used for controlling pests in the IPM system, could synergistically and negatively affect honey bee survival and significantly contribute to colony depopulation [26-30]. So far, the only registered treatment for Nosema disease is fumagillin, whose use is forbidden in Europe because it has no established Maximun Residue Level (MRL). With prolonged use of fumagillin, disease resistance to treatment has become an issue [31]. As a result, additional therapeutic options are urgently needed.

European honey bees, *Apis mellifera,* are the most important insect pollinator and are responsible for the pollination of one third of agricultural food crops in the world with an estimated value of $216/€153 billion per year [32]. For decades, nosema disease was exclusively attributed to *N. apis*, first described in 1909 [33]. In 2005, a natural infection of another microsporidia species, *N. ceranae,* which was first found in the Asian honey bee *Apis cerana*[34], was identified in *A. mellifera* colonies [35,36]. Subsequently, *N. ceranae* has become the predominant infection of two bee species [37-41] and has been implicated in honey bee Colony Collapse Disorder (CCD) [15,19,42-44], a malady that has decimated honey bee colonies across the U.S. and around the world [45-47]. The emergence of *N. ceranae* has changed the epidemiological pattern of honey bee nosemosis***,*** resulting in an urgent need for elucidating the genetic basis that defines the epidemiology and pathogenicity of the *Nosema* species.

Microsporidia genomes are highly reduced and compact in nature. The size of microsporidia genomes has been determined for sixteen microsporidia species mostly based on karyotyping analysis and EST surveys and varies considerably, ranging from 2.3 to 23 Mbp (reviewed in [48,49]). The first full genome sequence of a microsporidia species, *Encephalitozoon cuniculi,* marked a significant milestone in the study of microsporidia biology [50]. The genome organization and gene content of *E. cuniculi* represents the first evidence of microsporidia genomic reduction (2.9 Mb) and has provided considerable functional insight on the evolution of microsporidia-host relationships at the biochemical and molecular levels. Since then, seven additional microsporidia genomes have been sequenced [51-57].

In 2009, using the whole-genome shotgun (WGS) sequencing approach, we sequenced, annotated, and analyzed the complete genome of *N. cerana*e at 25X coverage [52], which represents a second full genome sequence of microsporidia. Like many microsporidia, *N. cerana*e has a highly reduced and compact genome with a draft assembly of 7.86 Mbp. A total of 2,641 putative protein-coding genes were identified based on computational analysis of the *N. cerana*e genome sequences. By comparing the *N. ceranae* putative protein-coding genes with those of fully sequenced *E. cuniculi* as well as with yeast, *Saccharomyces cerevisiae,* about 50% of *N. ceranae* genes appeared to be conserved among microsporidia. These microsporidia specific genes are of special interest for identification of potential virulence factors and elucidation of molecular mechanisms of intracellular invasion of these obligate intracellular parasites*.* We have now extended our efforts for genome-wide analysis of insect microsporidia to a second species, *N. apis* which is the first described microsporidia species of honey bees. In this report, we present a draft sequence of the *N. apis* genome and an annotation of the *N. apis* genome at the nucleotide and protein levels. We undertake comparative analyses of *N. apis* with its sympatric congener *N. ceranae* in order to identify candidates of species-specific coding sequences that may be associated with adaptation and virulence of the intracellular parasites. We hope that our genomic studies of honey bee microsporidia parasites will lead to greater understanding of the biology and pathogenesis of these difficult-to-treat fungal pathogens and contribute to the worldwide efforts to manage honey bee diseases and improve pollination services provided by honey bees.

## Results and discussion

### Species specificity of *N. Apis*

**Figure 1:**
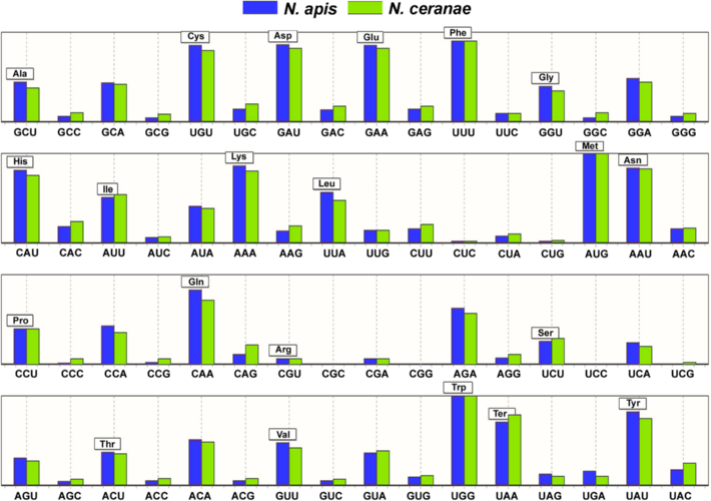
**Morphological and molecular confirmation of *N. apis species status. *(a)** Light micrograph of *N. apis* spore. Scale bar: 10 μM. **(b)** Agarose gel electrophoresis of PCR products showing the presence of a single *N. apis* infection in the DNA sample. The presence of a single *N. apis* infection in the DNA sample intended for 454 pyrosequencing was confirmed by species-specific PCR and further sequencing analysis.

Light microscopy revealed that fresh *N. apis* spores were oval shaped and varied in size, with a minimum of 5.15 μm and maximum of 7.415 μm in length, and a minimum of 2.99 μm and maximum of 4.836 μm in width (average 6.41 ± 0.74 x 3.36 ± 0.39 μm, N = 50) (Figure 1a). A single specific band was produced from the genomic DNA isolated from purified spores after PCR amplification with *N. apis* specific primers, and the sequence analysis confirmed the species specificity of the PCR fragment. No PCR product was generated when *N. ceranae* specific primers were added to the PCR mixture (Figure 1b).

### General features of *N. Apis* genome assembly

This Whole Genome Shotgun project has been deposited at DDBJ/EMBL/GenBank under the accession# ANPH00000000. The version described in this paper is the first version, ANPH01000000. The raw reads were submitted to the Sequence Read Archive (SRA) database with accession # SRA067788. There are 8,274,787 nucleotides grouped in 1,138 contigs, with an N_50_ contig size of 14,029 bp, and a mean contig size of 7,733 bp. These 1,138 contigs were placed into 554 scaffolds linked through paired reads with an N_50_ scaffold size of 24,309. The average number of contigs per scaffold was 2.05 with a minimum of 1 contig in a scaffold and maximum of 19 contigs in a scaffold. The scaffolds contain 584 intra-scaffold, or sequence, gaps with an estimated average length of only 74 bp. This large number of short gaps is consistent with the limitations of sequencing high-AT content genomes. Of the 1,138 contigs, 275 are larger than 10 Kbp and cover 63% of the genome. The average depth of coverage for non-repetitive contigs is 15X. Because of the surrogate-placement repeat handling of the Celera Assembler, the average coverage of all contigs, including repetitive regions of the genome, is 22X. Including both the total contig length and estimated gap lengths based on paired-reads, the genome of *N. apis* is estimated to be 8.5 megabases (Mb). Sequencing and assembly statistics are summarized in Table 1.

**Table 1:** ***N. apis *genome assembly statistics**

**Component**		**Value**
Scaffold		
	Number of Total Scaffold	554
	Minimum Scaffold Span	335
	Maximum Scaffold Span	154351
	Mean Span of Scaffolds	15447
	Scaffold N50	24309
	Total Bases in Scaffold	8.5 Mb
Contig		
	Number of Total Contigs	1,138
	Minimum Contig Length	97
	Maximum Contig Length	75980
	Mean Contig Length	7482
	Contig N50	14,029

### Contents of the *N. Apis* genome

The main features of the *N. apis* genome in comparison with *N. ceranae* are summarized in Table 2. The comparison of the size and contents of genomes indicated that the overall genetic structure, contents, coding capacity, and proteome complexity are very similar, reflecting the shared evolutionary history of both species. The search of the *N. apis* genome sequence assembly for repetitive DNA showed that the simple repeats and low complexity cover about 9.92% of the total sequence data. There were 7,789 tandem repeats identified with dinucleotide “AT” having the most abundant repeats dispersed in the *N. apis* genome. Three long terminal repeats (LTR) were identified. Three piggyback and three gypsy transposable elements were identified in the genome.

**Table 2:** **Main features of the *N. apis *genome in comparison with *N. ceranae***

	***N. apis***	***N. ceranae***
Size (Mb)	8.5	7.86
GC percentage (total genome) (%)	18.78	26
GC percentage in coding sequences (%)	25	27
GC percentage in 3rd position	13	18
tRNA genes (#)	62	65
Protein coding genes (CDSs)	2771	2614
Gene density (genes/kb)	0.326	0.33
Percent coding (%)	26.8	24.2

The *N. apis* genome is very AT rich, with an overall GC content of 18.78%, 7.2% lower than that of *N. ceranae*. The protein coding regions have significantly higher GC content (25%) compared to the overall GC content for the entire genome, indicating that GC content is not uniformly distributed across the genome and supporting the notion that GC content is higher in coding regions than in non-coding regions. The GC content also varies by codon position, with the 1st and 2nd positions of the coding regions having greater GC content than the third (synonymous) position.

**Figure 2:**
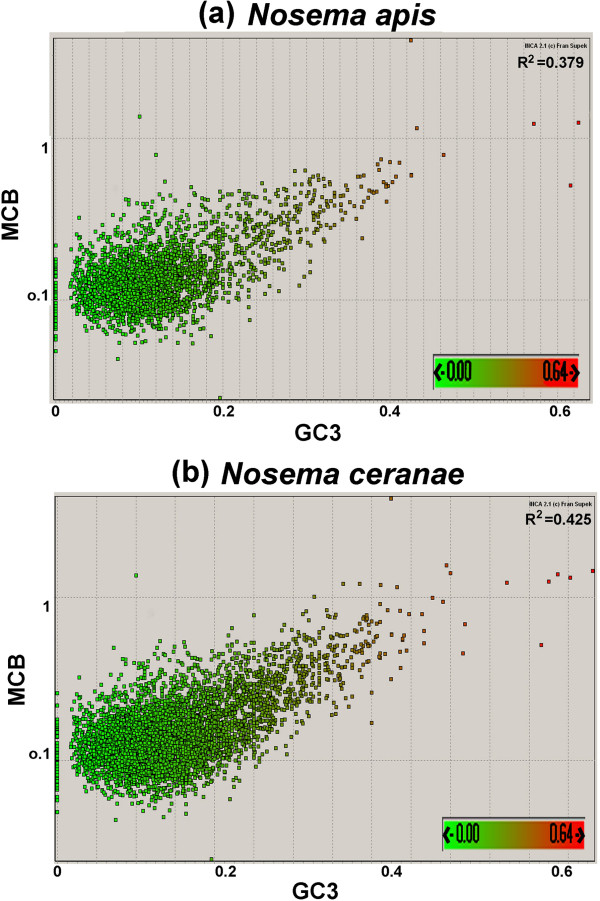
**Codon usage frequencies of *N. apis *(Blue) and *N. ceranae *(Green) gene groups.** Each bar represents the proportion of all codons encoding a given amino-acid that are the specified codon. The values are one by definition for the single-codon amino acids, tryptophan (Trp) and methionine (Met). Both *N. apis* and *N. ceranae* share similar optimal codons.

**Figure 3:**
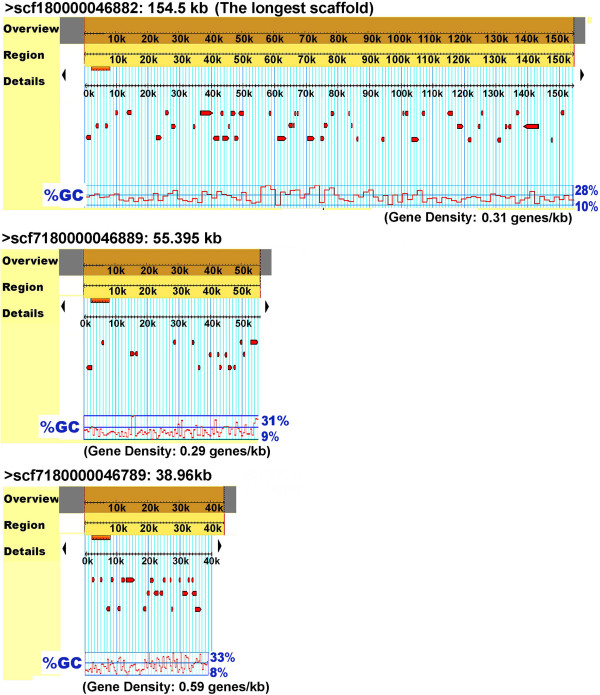
**Maximum-likelihood codon bias (MCB) analysis.** The GC content at the third sites of *N. apis* and *N. ceranae* genes was plotted against codon bias of genes of *N. apis* and *N. ceranae* genomes individually. MCB analysis reflects that the level of correlation between codon usage bias and GC content at the silent sites is higher in *N. ceranae* than in *N. apis.*

*N. apis* displays bias in its codon usage and has a pattern similar to that of *N. ceranae.* Both *N. apis* and *N. ceranae* have similarly preferred or optimal codons that have an A or T ending. However, codons ending with A or T are used more frequently in *N. apis* while codons ending with C or G are used more frequently in *N. ceranae* (Figure 2). The Maximum-likelihood codon bias (MCB) analysis showed that *N. apis* has a relatively lower level of correlation between GC content at the third position and the MCB (R^2^ = 0.379, ρ < 0.05), compared to *N. ceranae* (R^2^ = 0.425, ρ < 0.05) (Figure 3).

**Figure 4:**
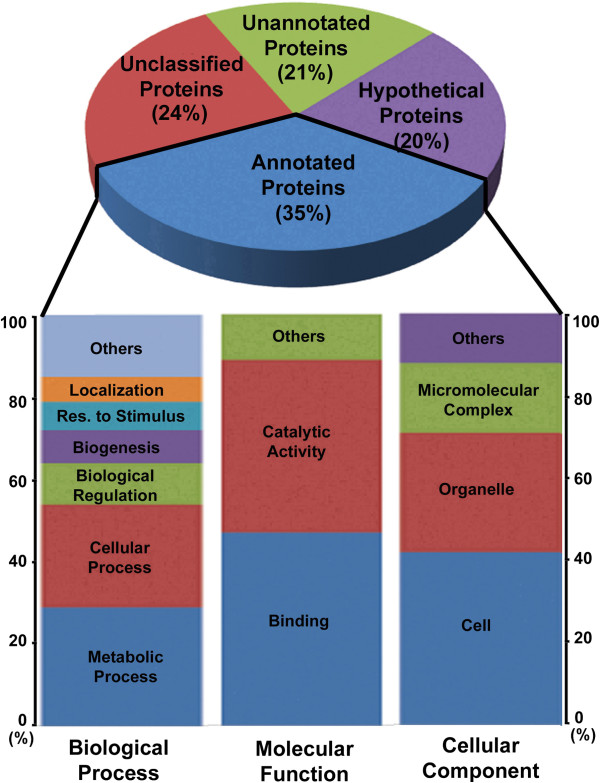
The genome browser screenshot showing six-frame translation and % of GC content on sequenced scaffolds.

The merged gene sets from the different programs resulted in a mixture of 2771 predicted protein coding regions (CDSs) made up of 2,159 (78%) intact and 612 (22%) intron interrupted protein-coding genes. The introns were inserted in random places within the genes and the genes containing introns fell into different functional groups. The consensus sequences “A(G/T)GT” at the donor site and “A(G/A)” at the acceptor site were identified in most of the intron-containing genes (Additional file 1: Figure S1). The density of genes across the genome is 0.33, which is similar to that for *N. ceranae*. However, no significant correlation between the length of scaffolds and the level of gene density was identified (Figure 4). Among the predicted CDSs, there are 2,684 complete genes and 87 partial genes that lack either a start or stop codon, or both. The length of CDSs averaged 274.88 amino acid residues per CDS.

**Figure 5:**
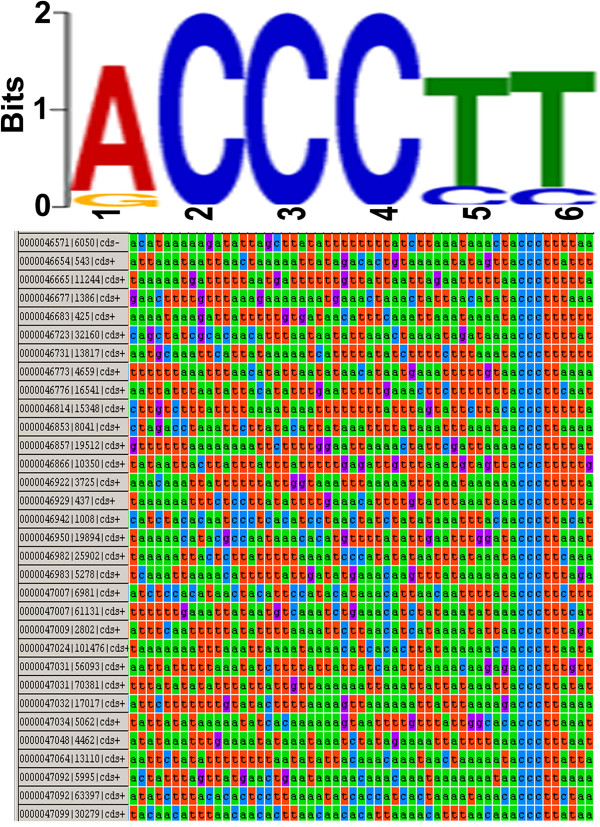
**Percentage of *N. apis *predicted genes as annotated proteins, hypothetical proteins, proteins without annotation and proteins without blast hit.** Annotated proteins share similarity with GO-annotated proteins. Unannotated proteins do not share sufficient similarity with GO- annotated proteins. Hypothetical proteins share similarity with previously published proteins of unknown function. Unclassified proteins have no sequence similarity with any sequence in public databases. The percentage for each group is given in parentheses. The annotated proteins were further classified into three GO categories: biological process, molecular function, and cellular component (Level 2). The y-axis indicates the percentage of a specific category of genes within the main categories.

Of these 2,771 CDSs, 2,105 (76%) had matches to the nr protein database (Threshold E value < 1e-03). Most of the sequences have an e-value between 1.0e-3 and 1.0e-25 (Additional file 2: Figure S2a). Among the 2,771 CDSs, 545 (20%) were assigned as conserved hypothetical proteins, 978 (35%) were assigned with GO terms, 582 (21%) were unannotated proteins and 665 (24%) had no match to the databases (Figure 5). The sequences without positive BLAST hits may be explained by a low similarity of the genes to those functionally similar genes in the database, or new genes that possess new functions in the parasite. The sequence similarity of *N. apis* within the database by Blast search ranges from 35% to approx 98% and peaks at 52% (Additional file 2: Figure S2b). The sequences of *N. apis* genes showed the most significant similarity to the sequences of *N. ceranae* followed by sequences of *E. intestinalis* and *E. cuniculi* (Additional file 2: Figure S2c). The majority of functional prediction of the coding sequences are obtained from the UniProt Knowledge Base (KB), a nonredundant protein database that includes PDS, UniProt, Swiss-Prot, TreMBL, and TAIR (Additional file 3: Figure S3a, 3b, and 3c). The *N. apis* reference names, gene prediction sources, the gene locations, coding strand, and conceptual translations are shown in a Gene-Finding File (GFF) formatted dataset (Additional file 4: Table S1). Table S1 also lists Blast-hit annotation, Enzyme code (EC), InterPro motifs, signal peptide and transmembrane motifs.

**Figure 6:**
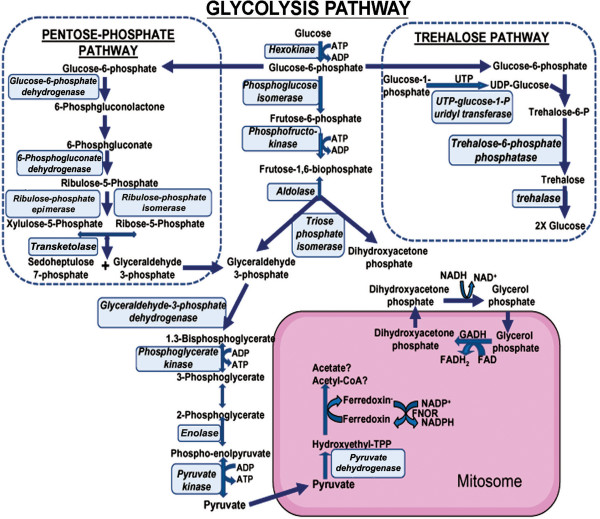
**Putative regulatory C-rich motif in upstream regions of start codons of *N. apis *genes.** Sequence logo of motif indentified by MEME and representative upstream sequence of start codon of *N. apis* genes showing the putative regulatory motif.

A search for motifs in the 100-bp upstream regions of start codons of *N. apis* genes showed an enrichment of TATA box motifs in the upstream of genes and also yielded three distinct MEME motifs. Of the three over-represented motifs, the “ACCCTT” motif conserved in *N. ceranae* and *E. cuniculi*[52] was particularly prominent (E = 1.14e-242), and was present in 37% of the predicted genes. The motif shows a preference for occurring predominantly at the ~15 bp upstream position of the start codon. The “ACCCTT” sequence logo and representative upstream sequences of start codon of *N. apis* genes are shown in Figure 6.

The predictions by SignalP and TMHMM showed that 399 proteins contained putative signal peptides with over 70% of the probability and significantly high C, S and Y scores and that 348 proteins contained putative transmembrane segments of some form with a score of equal or more than 0.3. Of 348 proteins that had membrane-spanning domains, 114 were predicted to contain an amino-terminal signal peptide. The signal peptides, and transmembrane motifs are also included in the dataset (Additional file 4: Table S1).

The combination of noncoding RNA (ncRNA) prediction programs and BLAST similarity search algorithms identified tRNA genes, rRNA gene clusters (16S, 23S and 5S), and small nuclear RNA (snRNA) genes. Both ARAGORN and tRNAscan-SE yielded similar results for tRNA prediction and showed a diversity of tRNA genes in the *N. apis* genome. There were 62 tRNA genes from ARAGORN and 58 genes from tRNAscan-SE as well as 16 tRNA-derived pseudogenes in tRNAscan-SE. By combining predictions from two tRNA prediction programs, there were 60 overlapping tRNAs with 42 distinct anticodons and gene lengths of 57 to 68 nucleotides. The 16S, 23S, and 5S rRNA genes were organized as a typical co-transcribed operon and arranged in order of 16S-23S-5S separated by two internal transcribed spacers (ITSs) in a tandemly repeated manner. The 16S, 23S, and 5S genes were identified in twenty-six scaffolds and four scaffolds contain a complete locus of 16S, 23S, and 5S genes. The sequence comparisons revealed polymorphisms among copies of rRNA genes and ITSs with the majority of polymorphisms found in the IGS region. The degree of intragenomic variability observed in 23S rRNA genes was greater than in 16S and 5S rRNA genes. While both 16S and 5S genes displayed 2% intragenomic variability, the average intragenomic variability observed it the 23S genes was about 10% with one 23S rRNA gene showing extreme divergence and sharing only 82% nucleotide sequence homology with the other 23S rRNA genes. While sequence alignment identified short conserved sequence motifs in ITSs, the intragenomic variability observed in ITSs was demonstrated not only by nucleotide substitutions but also by length variance across the genome.

The comparison of the 2,771 putative *N. apis* proteins with the 2,614 *N. ceranae* proteins revealed a broadly similar gene set shared between *N. apis* and *N. ceranae.* The reciprocal best BLAST search comparing the *N*. *apis* gene set and *N. ceranae* gene set yielded 1,356 one-to-one orthologs that were each other’s mutual best matches; thus, the orthologs corresponded to 49% of *N. apis* genes and 52% of *N. ceranae* gene sets. Of 1,356 ortholog pairs for both *N. apis* and *N. ceranae*, 137 orthologs had multiple hits in *N. apis*, suggesting the presence of paralogous proteins. The list of ortholog pairs in the genome of *N. apis* and *N. ceranae* can been found in Additional file 5: Table S2.

### Functional annotation of protein coding genes

**Figure 7:**
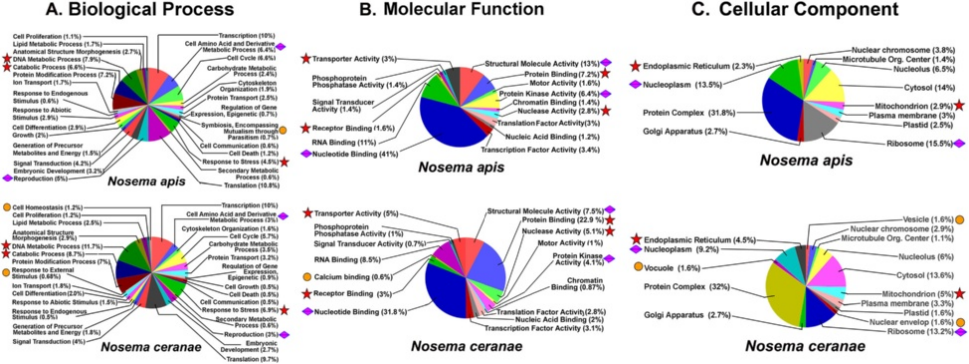
**Homologous proteins of *N. apis *and *N. ceranae *in core pathways of carbon metabolism.** Enzymes encoded by both *N. apis* and *N. ceranae* genomes are boxed. The conservation of enzymes in carbon metabolic pathways suggests that carbohydrate metabolism is the main process responsible for energy generation in *Nosema*.

Among sequences that were assigned with gene ontology (GO) functional classification terms, 103 out of 978 sequences were further annotated with Enzyme Commission (EC) codes. Annotated sequences were further mapped to 50 different biological pathways. The KEGG pathway map revealed the presence of a large number of *N. apis* sequences involved in nucleotide metabolism including purine metabolism and pyrimidine metabolism. Genes encoded all key enzymes involved in glycolysis, the pentose-phosphate pathway, and the trehalose pathway identified in *N. ceranae* were also found in *N. apis*, indicating that carbohydrate metabolism is the main process responsible for energy generation in *Nosema* (Figure 7). *N. apis* also contained enzymes participating in amino acid and lipid metabolism. It was not unexpected that *N. apis* lacked genes encoding enzymes involved in electron transfer chains and the tricarboxylic acid (TCA) cycle, which corroborates previous findings in other microsporidia and indicate a strong host dependence for energy production. The KEGG pathway map also revealed that *N. apis* harbored genes for aminoacyl-tRNA biosynthesis, a process that is essential for accurate translation of genetic information, and genes required for biosynthesis of secondary metabolites and microbial metabolism that are involved in the parasite’s growth, survival, and fecundity.

**Figure 8:**
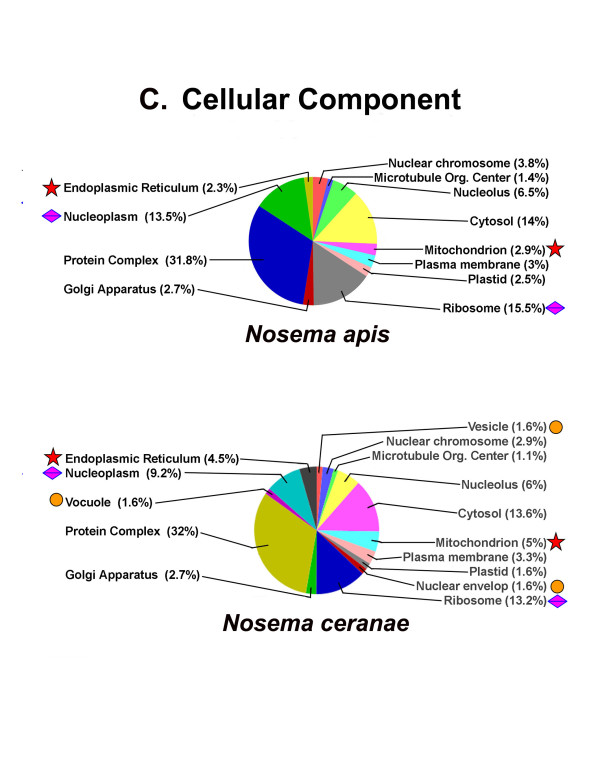
**Distribution of enriched functions in A) Biological Process (BP), B) Molecular Functions (MF) and C) Cellular Component (CC) by *N. apis *and *N. ceranae*.** For each panel, the pie displays the distribution of annotated sequences (%) by BP, MF, or CC in both *N. apis* and *N. ceranae* datasets. The stars and diamond shaped squares mark more than 2% difference of enriched GO terms between two species. The stars indicate specific GO terms that are significantly more prevalent in *N. ceranae* than in *N. apis* by functional enrichment analysis. The diamond shaped squares indicate specific GO terms that are significantly more prevalent in *N. apis* than in *N. ceranae* by functional enrichment analysis. The cycles indicated that GO are unique to either one species of *Nosema.*

Of protein sequences with assigned GO terms, most sequences have between 1 and 6 GO terms annotated. Annotation results and distribution, GO-level distribution, number of GO-terms for *N. apis* sequences with length (x-axis), annotation score distribution; and percentage of *N. apis* sequences with length (x-axis) annotated are shown in Additional file 6: Figure S4a, 4b, 4c, 4d, 4e and 4d. Of the proteins that had matches to the nr protein database, the most abundant protein class is the binding proteins. Other highly abundant proteins include intracellular transport proteins, ribosomal proteins, and proteins involved in major functional categories such as RNA processing, transcription and translation initiation and regulation, post-traslational modification and energy production. Among GO terms of molecular function, “Binding” was the most predominant group. Of GO terms identified for biological process, “metabolic”, was the most dominant term with 18 subcategories related to the metabolic process. Of categories enriched in the cellular component, protein complex, intracellular membrane-bounded and non-membrane bounded organelles were the largest groups (Figure 8A, 8B, and 8C).

Functional analysis showed that *N. apis* harbored a highly reduced mitochondrial homologue, the mitosome, and encoded a number of putative proteins that are candidate constituents of this mitosome. In total, seventeen genes encoding mitosomal proteins including ATP-binding cassette (ABC) transporter complex, sulfate transporter family protein, NADPH oxidoreductase, ISCU, hydrolase, heat shock protein 70, cation efflux protein zinc transporter, glycerol-3-phosphate dehydrogenase, exportin, and pyruvate dehydrogenase E1 (PDH) were identified from the complete genome sequence of *N. apis.* Consistent with the finding in *E. cuniculi*, the genes encoding PDH El alpha and beta subunits were found in *N. apis*. However, PDH E2 and E3, which are components of the PDH complex, are absent. This suggests that while PDH provides evidence of mitochondria-derived metabolic activity, the function of PDH in microsporidia may be different from that in other eukaryotes, where it catalyzes the decarboxylation of pyruvate to acetyl-CoA and CO_2_. The identified mitosomal proteins clustered into different functional groups, including iron-sulfur (Fe-S) cluster assembly and export, transporter, chaperones and co-chaperones, tubulin and motor proteins, metabolic processes and anti-oxidative stress. The proteins involved in Fe-S cluster assembly are essential to mitochondrial function under oxygen-limiting conditions and are the most prominent functional group within the predicted mitosomal proteins. The putative mitosomal proteins identified in *N. apis* are listed in Table 3.

**Table 3:** ***N. apis s*equences that show homology to annotated and experimentally verified pathogenicity and virulence genes of microsporidia species**

**Product/Gene**	**Sequence ID**	**Function**	**Homologous to *N. ceranae***	**Organism whose annotated sequence has top BlastP hit**	**Reference**
**1) Polar Tube Protein**					
PTP2	NAPIS-00435		Yes	*N. bombycis*	[77]
PTP3	NAPIS-01921	Host cell invasion	Yes	*N. bombycis*	[77]
PTP3	NAPIS-01922		Yes	*N. bombycis*	[77]
**2) Spore Wall Protein**					
SWP12	NAPIS-00521		Yes	*N. bombycis*	[78]
SWP25	NAPIS-00461		Yes	*N. bombycis*	[79]
	NAPIS-00514	Structural capacity &	Yes	*N. bombycis*	[79]
	NAPIS-01092	host cell adhesion	Yes	*N. bombycis*	[79]
SWP26	NAPIS-00203		Yes	*N. bombycis*	[80]
SWP32	NAPIS-00269		Yes	*N. bombycis*	[78]
**3) Endochitinase**					
	NAPIS-02055	Spore attachment	Yes	*E. hellem*	[56]
	NAPIS-02138		No	*E. hellem*	[56]
**4) Chitin synthase**					
	NAPIS-01775	Spore attachment	Yes	*E. intestinalis*	[53]
	NAPIS-02139	& pathogenicity	Yes	*E. hellem*	[56]
**6) Mitogen-activated protein kinase**					
	NAPIS-01928	Pathogenicity Regulation	Yes	*E. intestinalis*	[53]
**7) ATP/ADP Translocase**					
	NAPIS-01593		Yes	*E. cuniculi*	[50]
	NAPIS-01593	Energy parasitism	Yes	*E. cuniculi*	[50]
	NAPIS-02128		Yes	*E. cuniculi*	[50]
	NAPIS-02287		Yes	*E. cuniculi*	[50]
**8) Transferase**					
	NAPIS-00457		No	*E. hellem*	[56]
	NAPIS-00459	Pathogenicity	Yes	*E. cuniculi*	[50]
	NAPIS-00459	regulation	Yes	*E. intestinalis*	[53]
	NAPIS-00578		No	*E. hellem*	[56]
	NAPIS-00596		Yes	*E. hellem*	[56]
**9) Splicing machinery**					
	NAPIS-00564		Yes	*E. intestinalis*	[53]
	NAPIS-00654	Pathogenicity	Yes	*E. intestinalis*	[53]
	NAPIS-01012	regulation	Yes	*E. hellem*	[56]
	NAPIS-02190		Yes	E. romaleae	[56]
** 9) Putative secretive proteins (**399 proteins listed in Additional file 4: Table S1)					

Virulence factors are molecules expressed and secreted by parasites that are keys for causing disease in the host as the parasites enter into and exit out of host cells, and inhibit certain host functions. Genome analysis of *N. apis* has identified genes encoding a wide array of potential virulence determinants. These potential virulence factors of *N. apis* include genes encoding 1) polar tube proteins defining structure and invasion mechanisms; 2) spore wall and anchoring proteins involved in host tissue recognition and the initiation of host cell invasion; 3) endochitinase and chitin synthase involved in spore-host cell attachment; 4) proteins involved in pathogenicity regulations and energy parasitism, 5) components of the spliceosome machinery that plays a major role in the generation of proteomic diversity and proteins regulating splicing activity, and 6) putative secretive proteins that are involved in interactions with the host and are essential for modulation of host immunity towards successful completion of the parasite lifestyle in the host (Table 3).

### Functional conservation and divergence of protein sequences between *N. Apis* and *N. Ceranae*

While putative proteins of *N. apis* share significant sequence homology and functional similarity for enriched GO terms with *N. ceranae*, the comparison of the GO terms based on Fisher’s exact test after a false discovery rate correction (p < 0.05) displayed some differences between the two species. Comparison of biological processes (Figure 8A) showed that genes involved in the developmental process, organismal development, signaling, kinase activity, translational activity were on the same level of abundance for both species. However, genes that are functionally linked to cell amino acid derivative metabolic process and reproduction were significantly more common in *N. apis* than in *N. ceranae.* In contrast, genes involved in metabolic processes and stress response were greatly expanded in *N. ceranae*. The protein transport genes were also found to have greater representation in *N. ceranae* than in *N. apis. N. apis* and *N. ceranae* also displayed different protein profiles responsive to stress and stimuli. Genes responsive to stress were over represented in *N. ceranae* compared to *N. apis,* while *N. apis* contained a slightly greater number of genes responding to abiotic stimuli. Both species had about the same number of genes related to responses to endogenous stimuli. While cell homeostasis and response to external stimulus genes were only identified in *N. ceranae,* symbiosis and encompassing, mutualism through parasitism genes were unique to *N. apis*. The genes related to response to external stimuli were present only in *N. ceranae* and absent in *N. apis.* These gene families may therefore represent species-specific proteins. For molecular function GO terms, a number of differences were observed between the two *Nosema* species (Figure 8B). Structure molecule activity proteins that contribute to the structural integrity of a complex or assembly within or outside a cell were found significantly reduced in *N. ceranae*, compared to *N. apis*. The calcium binding protein that was not in *N. apis* was present in *N. ceranae*. An enrichment test of cellular components showed that genes encoding nucleolus, Golgi apparatus, cytosol, nuclear chromosome, mitochondrion and plasma membrane were at approximately similar levels of abundance for both species. However, intracellular membrane-bounded organelles including mitochondrion and endoplasmic reticulum were overrepresented in *N. ceranae* relative to *N. apis*. In contrast, nucleoplasm was significantly more common in *N. apis* than in *N. ceranae*. The ribosome was also found to be more represented in *N. ceranae* in relation to *N. apis*. The vacuole and vesicle proteins were present in *N. ceranae* but absent in *N. apis*, suggesting species-specific genes involved in structure and function of cellular components.

## Conclusions

*Nosema* disease is regarded as one of the most destructive and widespread adult honey bee diseases in existence and has become a serious problem in the beekeeping industry worldwide because of an alarming increase in the disease’s prevalence as well as its association with the widespread collapse of honey bee colonies. The sequencing and annotation of the *N. apis* genome provide a comprehensive overview of genetic content, structure and organization of the parasite and give some interesting insights into the complex biological and molecular processes of the parasite. The comparison of the *N. apis* genome to its sympatric congener, *N. ceranae,* provides an opportunity to define a subset of genes and their associated pathways that may be responsible for the pathogenesis of *Nosema* disease and identify potential virulence factors that could be used as potential drug targets and for the development of novel antimicrobial agents. The identification of an overrepresented (ACCCTT) motif in the upstream promoter regions of 37% of *N. apis* genes led us to wonder if these genes have similar patterns of promoter activity and are regulated by the same transcriptional factors. Post-genomic experimental investigations are warranted to identify possible transcriptional factors and to determine the roles of the motif in the gene expression profile of *Nosema*, parasitic processes of development and host invasion. The transcription factors may offer an attractive opportunity to exploit RNAi-based targets for therapeutic intervention of *Nosema* disease.

Genomic data of *N. apis* and *N. ceranae* showed that overall genetic structure, contents, coding capacity and proteome complexity of *N. apis* and *N. ceranae* are similar, suggesting a shared evolutionary history for the two species. Previous studies have shown that variability in codon usage is correlated strongly with GC content, particularly at the third codon position (GC3), and also that highly expressed genes exhibit very high levels of codon bias. The evolution and functional significance of the genome variation in GC content and codon usage bias have been widely reported between organisms and also within a genome; however, the underlying causes of these variations remain poorly elucidated and there has been a long debate over whether these variations are selected or neutral traits [58]. It is hard to define whether selection plays a role in codon usage bias in *N. apis* with observed weak correlation between codon usage bias and the GC content at the third site (GC3). The slight difference in correlation of genomic GC content and codon usage between *N. apis* and *N. ceranae* may reflect an evolutionary divergence or may simply be due to gene length effects of the two species.

The large number of orthologous protein groups shared by *N. apis* and *N. ceranae* and similar biochemical pathways encoded by *N. apis* and *N. ceranae* suggests that the core genes and their associated functions have been retained in the genus *Nosema*. The inferences of biological process, molecular function and cellular component categorizations based on predicted gene products, however, distinguish the two species. Although *N. apis* and *N. ceranae* lack the sophisticated biosynthetic pathways that are necessary for energy production, biochemical analyses showed that ATP transporter proteins are enriched in both parasites, largely reflecting their host-dependent intracellular lifestyle. The key enzymes involved in energy transporter activity and metabolic process are more represented in *N. ceranae* than *N. apis*, suggesting that *N. ceranae* may have a greater capacity for biosynthesis and better ability to obtain energy from its host, compared with *N. apis*. Although *N. ceranae* is a comparatively recent introduction into populations of European honey bees, *N. ceranae* is the more prevalent of the two *Nosema* species in honey bees and appears to be replacing *N. apis* in some populations of *A. mellifera*[37,43,59,60]. The high survival capacity of *N. ceranae* would allow the parasite to consume the host for its own growth and multiplication and therefore reduce the host’s fitness over time but generally not kill it. In contrast to the distinctive disease symptoms caused by *N. apis,* such as dysentery and crawling behavior, *N. ceranae* infection is generally not associated with external symptoms. Instead, infected colonies have been found to decline gradually and collapse over a period of time. The differences in host pathogenicity between *N. apis* and *N. ceranae* may be, in part, a result of their differences in molecular function and biological process. The novel sequences of *N. apis* without detectable homologs in *N. ceranae* and vice versa suggests that there has been gene shuffling along the evolutionary pathway separating *N. apis* and *N. ceranae* from their common ancestor.

The success of the parasite also relates to its ability to respond to stress and endogenous stimuli within hosts. The result that proteins involved in response to stress and endogenous stimulus were more represented in *N. ceranae* than *N. apis* suggests that *N. ceranae* may have a better ability to survive under stressful conditions such as host immune functions. Alternatively, *N. apis* may be able to sense and respond rapidly to abiotic stimuli such as environmental changes, as categories related to the response to abiotic stimuli were more represented in *N. apis* than in *N. ceranae*. This assumption is supported by recent findings that *N. ceranae* and *N. apis* responded differently to decreasing fumagillin levels [31] and that *N. apis* was more resistant to cold temperatures than *N. ceranae*[61].

Discovering virulence factors is a key to understanding microsporidia pathogenesis and their interactions with their host and for identifying targets of novel drugs. The data from the genome sequences of *N. apis* as well as *N. ceranae* have allowed us to identify a number of putative virulence factors. For example, host tissue recognition and polar tube extrusion for initiating cell invasion are important factors in the pathogenesis of microsporidia parasites. Parasite spore wall and polar tube proteins that are essential for host cell specificity and cell invasion processes therefore could be potential targets for development of antigen-based diagnostic tests, antimicrobials and siRNA molecules. The proteins secreted from cells into the extracellular space mediate important parasite-host interactions and therefore are also interesting targets for innovative therapeutics. Future experimental evaluation of these virulence factors of microsporidia through molecular manipulations such as RNA interference (RNAi) [62] should provide valuable insight into their usefulness in drug development.

Although they share some morphological, biological and genetic features, as well as invasive strategies, the genome sequences of *N. apis* and *N. ceranae* revealed that these microsporidia species are highly diversified and that not all microsporidia genomes are characterized by reductions in size and complexity. *Nosema* genomes appear to be much less compact than the smallest microsporidia genomes, such as *E. cuniculi*, providing new insight into the adaptive evolution caused by the host-parasite relationship across the microsporidan groups. The availability of draft sequences of the *N. apis* genome is a significant addition to the rapidly expanding body of microsprodian genomic data which has been improving our understanding of eukaryotic genome diversity and evolution in a broad sense.

## Methods

### Bee sample collection and spore purification

Adult worker bees infected with *N. apis* were collected from an apiary infected exclusively with *N. apis* in Beaverlodge, Alberta, Canada in 2007. The species status of the *Nosema* infection was determined by a combination of morphological and molecular methods. In order to purify spores of *N. apis* from the infected bees, alimentary tracts were individually removed by grasping the stinger with forceps and pulling it out with gentle traction. The infected alimentary tracts were homogenized in 3 ml sterile water using a conical ground-glass homogenizer with a rough grinding surface and filtered through a Corning (Lowell, MA) Netwell insert (24 mm diameter, 74 mm mesh size) to remove large-sized tissue debris. The filtered suspension was centrifuged (Rotor: Sorvall RC-5B) at 3,000 g for 5 minutes to pelletize the spores. The resuspended pellet was then purified on a discontinuous Percoll (Sigma-Aldrich, St. Louis, MO) gradient consisting of 10 ml each of 30%, 60%, and 90% Percoll solution by overlaying the spore suspension onto the gradient and centrifuging at 8,000 g for 10 minutes. The pelleted spores were further purified with 30 ml of 100% Percoll solution by centrifuging at 5,000 g for 2–3 minutes to separate the spores and lighter cellular debris into the top and bottom layers, respectively. The spore-containing supernatant was collected and diluted with water in the ratio of 1:1 and centrifuged at 8,000 g for 10 minutes to pellet the spores. The purity and size of spores were examined under a light microscope (Nikon, Eclipse TE 300) and photographed with a Nikon Digital Camera (DXM 1200).

### Genomic DNA isolation and N. Apis species confirmation

The genomic DNA was isolated using CTAB (hexadecyltrimethylammonium bromide) extraction buffer following spore disruption. Approximately 10^6^ *N. apis* spores were suspended in 500 ml CTAB buffer (100 mM Tris–HCl, pH 8.0; 20 mM EDTA, pH 8.0; 1.4 M sodium chloride; 2% cetyltrimethylammonium bromide, w/v; 0.2% 2-mercaptoethanol) and broken by adding 500 mg of glass beads (425–600 mm, Sigma-Aldrich, St. Louis, MO) into the tube and disrupting the mixture at maximum speed for 3–5 minutes using a FastPrep Cell Disrupter (Qbiogene, Carlsbad, CA). The mixture was then incubated with proteinase K (200 mg/ml) at 55°C for five hours and treated with chitinase (0.4 U) with an extended incubation period of 16 hours at 55°C. The genomic DNA was twice extracted in an equal volume of phenol/chloroform/isoamyl alcohol (25:24:1), followed by a single extraction in chloroform. The purified DNA was precipitated with isopropanol, washed in 70% ethanol, and dissolved in 100 μl sterile water. The concentration and purity of the DNA were determined by spectrophotometric absorption at 260 nm, and ratios of absorption at 260 nm and 280 nm.

The *Nosema* species specificity of extracted genomic DNA was confirmed by a PCR assay prior to 454 pyrosequencing. Primers specific to *N. apis* and to *N. ceranae* were used individually in the PCR-mediated amplification of genomic DNA isolated from purified spores. The sequences of *N. apis* primers that amplified 269-bp fragment of the 18S rRNA were as follows: apisF 5′- CCATTGCCGGATAAGAGAGT -3′ and apisR 5′- CCACCAAAAACTCCCAAGAG -3′. The PCR reaction mixture, thermal conditions, and sequences of *N. ceranae* primers used were as previously described [41]. The amplified PCR products were purified using the Wizard PCR Prep DNA Purification System (Promega, Madison, WI) and the nucleotide sequence identity of the PCR fragments was determined using a Basic Local Alignment Sequence Tool (BLAST) homology search.

### Library construction, sequencing, and assembly

Although the high% AT vs.% GC ratio of this genome poses challenges for all high-throughput sequencing platforms, a successful sequencing library was constructed, sequenced and assembled through protocol optimizations. The 454 Titanium sequencing platform offers an ideal combination of throughput and long read length to enable de novo sequence assembly of whole genomes. A paired-end library was constructed according to the manufacturer’s protocol, with modifications and optimizations to enable robotic automation of crucial steps and manipulation of limited, high-% AT starting material. An initial sample of 500 ng DNA was sheared using a Covaris E210 high-performance ultra-sonicator. Following adaptor ligation and double AMPure SPRI bead-based size selection, the resulting library had an average fragment size of 2,072 bp with a standard deviation of 208 bp. This library was sequenced on a full two-region 454 Titanium run resulting in 1,070,575 passed-filter sequence reads with an average quality-trimmed length of 304 bp. During data quality control procedures, 126,461 reads, or 11.8% of the total, were eliminated as PCR-induced duplicates. This represents a typical duplicate read percentage for the 454 sequencing platform. An additional 10% of the reads were eliminated as too short for assembly or containing an inconsistent or partial paired-end linker sequence. Of the remaining reads, 360,731 were bisected at the linker to form successful pairs, while 475,944 remained as unpaired shotgun reads.

These data were assembled using the Celera Assembler (http://sourceforge.net/projects/wgs-assembler/) version 6.1 using the CABOG module, an overlap-layout-consensus sequence assembler that has been recently optimized for use with 454 Titanium paired-end sequence data. The Celera Assembler uses a stringent algorithm that heavily weighs paired-end read constraints and favors shorter, higher-confidence contigs over longer, potentially misassembled contigs. This approach ensures a highly accurate result with minimal mis-assembly.

### Repetitive element identification

Genome analysis of *N. ceranae* showed the presence of a large proportion of the repetitive elements which could interfere with the accuracy of de novo gene prediction. We therefore used RepeatMasker (http://www.repeatmasker.org), Tandem Repeat Finder [63], and LTR Finder [64] in an ab initio manner with the default settings to identify the repetitive elements. Repetitive elements and low complexity DNA sequences in the *N. apis* genome were then masked for elimination. To avoid the incorrect identification of repetitive elements and low complexity from DNA sequences, manual inspection of the list of repeats was carried out. The repeats that had significant Basic Local Alignment Search Tool (BLAST) hits to the NCBI protein database (E-value cutoff of 1.0E-5) were kept for subsequent gene prediction and annotation.

### Protein-coding gene prediction and regulatory motif identification

After the exclusion of a few contaminating honey bee host sequences identified through BLASTN searches against the bee genome, a combination of two programs was used for identification of protein-coding sequences (CDSs) in the masked *N. apis* genome: GeneMark.hmm.ES (version 3.0) using non-supervised training procedures and Augustus trained with *E. cuniculi*, and *S. cerevisiae*. The GeneMark.hmm [65] and AUGUSTUS [66] programs for eukaryotic genomes access the coding potential of DNA sequence based on Hidden Markov Models (HMM). Next, outputs of supervised gene prediction based on sequence similarity to reference organisms (extrinsic method) and *ab initio* gene prediction relying on intrinsic features of the DNA sequence (intrinsic method) were merged and reconciled with other gene models. The CD-HIT-2D [67] was used for identifying overlapping and unique sets of proteins from different gene prediction programs. For overlapping predictions produced by different programs, the prediction with BLAST evidence was kept. If the overlapping predictions by different programs did not have BLAST evidence or if they all had BLAST evidence, the longest prediction was chosen. To verify the correct prediction of protein-coding genes, all inferred genes larger than 50 aa were further compared across an NCBI non-redundant protein database using BLASTP program with default parameters. Frame-shifts and point mutations were detected and corrected where appropriate. Candidates with a protein identity match ≥ 30%, and BLASTP value ≤ 10–5 were retained as protein-encoding gene candidates.

Nucleotide composition and synonymous codon usage of protein coding genes were investigated using the program INCA2.0 by [68]. The maximum-likelihood codon bias (MCB) was used as a measure for codon usage bias where the contribution to the index of the bias of each amino acid is weighted by an estimation of the likelihood of occurrence of bias on each amino acid [69]. The relative synonymous codon usage (RSCU) of individual codons between *N. apis* and *N. ceranae* were compared. Putative transcriptive regulators were searched by identifying over-represented motifs in the 100-bp upstream region of the start codon of each protein-coding gene. The MEME 3.0 software program [70] was used for the motif identification.

### Signal peptide and transmembrane region prediction

The N-terminal amino acid sequence of predicted protein from this study was analyzed for the presence of putative signal peptides using the SignalP 3.0 program [71], and a neural network and HMM trained on eukaryotes. The transmembrane regions of predicted protein were identified with TMHMM 2.0, a HMM based transmembrane helix prediction program. The architecture of the hidden Markov model and the neural network algorithm was applied for prediction of the signal peptide and transmembrane region of each translated protein. The default parameters were used for both programs.

### Non-coding RNAs

tRNA genes were detected using two tRNA identification programs, tRNAScan-SE version 123 (Lowe and Eddy, 1997) and ARAGORN [72] at the default settings. The tRNAScan-SE search was based on tRNA training data from a mixed general tRNA model. The ARAGORN search was based on the homology of recognized tRNA consensus sequences by specifying a yeast nuclear genome (NC_001133-NC_001148) and the ability of input data to form a base-paired cloverleaf to predict tRNA secondary structure. Two programs are also able to distinguish tRNA pseudogenes from true genes with high sensitivity.

Ribosomal genes coding rRNA in a full genome sequence were identified using both RNAmmer 1.2 server [73] with the default parameters and BlastN searches. Matches were defined as those sequences having greater than 90% similarity and greater than 90% of the query length. Small nuclear RNAs (snRNA) were also identified by BlastN searches. rRNA sequences were annotated based on homology to previously published rRNA sequences deposited in GeneBank.

### Ortholog and paralog identification

The possible orthologs of *N. apis* in *N. ceranae*, *E. cuniculi* or *S. cerevisiae* genomes were predicted by searching for the best reciprocal BLASTP matches with InParanoid [74]. In each of the between-species comparisons, we aligned protein sequences from one species to protein sequences from the other, and corresponding best matches were then aligned back to the original species. On the basis of all of the possible between-species sequence pairs, we reported orthologs and in-paralogs that had multiple hits in the same genome with a bit score cutoff of 40 and an aligned region larger than 50% of the longer query sequence in each pair. We also performed bootstrap analysis to obtain confidence for each ortholog identified. Gene duplications were identified with a focus on proteins in the data set that had a better match within the *N. apis* genome they reside (excluding the self match) when compared against all other genomes used in the study. In order to avoid assigning paralogs to ortholog pairs, the top hit had to have an E-value 10^5^ times lower (more significant) than the next best hit. In addition, the complete genome sequence of *N. apis* was aligned individually to the complete genome of *N. ceranae* to find the corresponding orthologous blocks in the genomes and to illustrate homologs between genomes by using the Mauve 2.2. [75].

### Protein domain functional annotation

The predicted proteins were analyzed using the BLASTP algorithm to search for homolog sequences and functionally annotated by gene ontology (GO) terms, InterPro terms (Conserved patterns in sequences, InterProScan, EBI), enzyme classification (EC) codes, and metabolic pathways (Kyoto Encyclopedia of Genes and Genomes, KEGG) using the Blast2GO (B2G) software suite v2.4.2 (http://www.BLAST2go.org, [76]. The functional annotation in B2G was performed using the following steps: 1) performing BlastP to final homologous sequences to the query set; 2) mapping to collect gene ontology terms (GO) associated to the hits after a BLASTP search; 3) selecting GO terms from the GO pool collected in the mapping step and assigning them to the query sequence; 4) grouping the descriptions into ‘GOslim” categories for biological process, molecular function, and cellular component, and 5) performing Enzyme code and InterPro annotations support language by GO terms. Finally, an enrichment analysis was performed to assess the differences in functional classes between *N. apis* and *N. ceranae* using one-way Fisher’s Exact Tests. The default settings of B2G were used in every annotation step. The false discovery rate (FDR) at level α = 0.05 was applied for controlling procedures. Gene product names are assigned based on sequence homology to known genes in NCBI databases. The putative proteins without GO annotation were classified as “hypothetical protein with no significant database hit”.

### Ethics statement

The apiaries for bee sample collection are the property of the USDA-ARS Beltsville Bee Research Laboratory, MD, USA and the Agriculture & Agri-Food Canada, Beaverlodge Research Farm, Beaverlodge, AB, Canada; therefore, no specific permits were required for the described studies. Studies involved the European honey bee (*Apis mellifera*), which is neither an endangered nor protected species.

## Competing interests

The authors declare that they have no competing interests.

## Authors’ contributions

YPC and JDE conceived and designed the study; YPC and MH performed the analysis; YPC, YZ, XL, LLT, DLS, XHL, SKH, HQZ, XZ, and XHY analyzed the data; YPC, JDE, JSP, SFP and APM contributed reagents/materials/analysis tools; YPC and LLT wrote the paper. All authors read and approved the final manuscript.

## Supplementary Material

Additional file 1: Figure S1 Exon–intron junctions in most of the intron-containing protein-coding genes of N. apis. The consensus sequences “A(G/T)GT” at the donor site and “A(G/A)” at the acceptor site were indicated by arrows separately. Click here for file

Additional file 2: FigureS2 Characteristics of homology searches of *N. apis* protein-coding genes against the non-redundant protein sequences (nr) at NCBI using Blastp. **(a)** E-value distribution of the top BLAST hit for each unique sequence with a cut-off E-value of 1.0e-3. The most sequences have an e-value between 1.0e-3 and 1.0e-25; **(b)** Similarity distribution of the top BLAST hit for each unique sequence. The sequence similarity of *N. apis* with database by Blast search ranges from 35% to approx 98% and peaks at 52%; and **(c)** Top-species distribution of the top BLAST hit for each unique sequence. The sequences of *N. apis* sequences showed the most significant similarity to the sequences of *N. ceranae* followed by sequences of *E. intestinalis* and *E. cuniculi.* Click here for file

Additional file 3: Figure S3 Mapping of *N. apis* protein-coding genes to GO terms associated to Blastp hits. **(a)** Evidence code distribution for BLAST hits. The evidence code distribution for BLAST hits chart shows an overrepresentation of Inferred Electronic Annotation (IEA), followed by Inferred by Direct Assay (IDA); **(b)** Evidence code distribution for individual sequences. The highest evidence code for the individual sequences was through Inferred Electronic Annotation (IEA), second by Inferred by Direct Assay (IDA) and third by Inferred by Mutant Phenotype (IMP); **(c)** Mapping database sources. The majority of *N. apis* genes are obtained from the UniProt Knowledge Base (KB), a nonredundant protein database that includes PDS, UniProt, Swiss-Prot, TreMBL, and TAIR. Click here for file

Additional file 4: Table S1 Predicted coding genes and annotations. Click here for file

Additional file 5: Table S2 Complete list of the ortholog pairs in the genome of *N. apis* and *N. ceranae.* Click here for file

Additional file 6: Figure S4 Functional assignment terms to query sequences from the pool of GO terms gathered in the mapping step. **(a)** Annotation results. Of CDSs of *N. aps* with blast hit, 545 were assigned as conserved hypothetical proteins. The subsequent mapping assigned GO terms to 978 positive BLAST hit sequences. **(b)** Annotation distribution; Most sequences have between 1 and 6 GO terms annotated; **(c)** GO-level distribution. *N. apis* sequence GO terms representation for biological process (BP), molecular function (MF) and cellular component (CC) ontologies. The mean GO-level is 5.749 and 5155 annotations could be assigned; **(d)** Number of GO-terms for *N. apis* sequences with length (x). The length of most GO term annotated *N. apis* sequences were are in the 80–700 bp range; **(e)** Annotation score distribution; and **(f)** Percentage of *N. apis* sequences with length (x) annotated. Click here for file
