# Supplementary figures and images for: Genome sequencing and comparative genomics of honey bee microsporidia, Nosema apis reveal novel insights into host-parasite interactions
Source: BMC Genomics. 2013 Jul 5;14:451. doi: 10.1186/1471-2164-14-451 (PMC3726280; doi:10.1186/1471-2164-14-451)

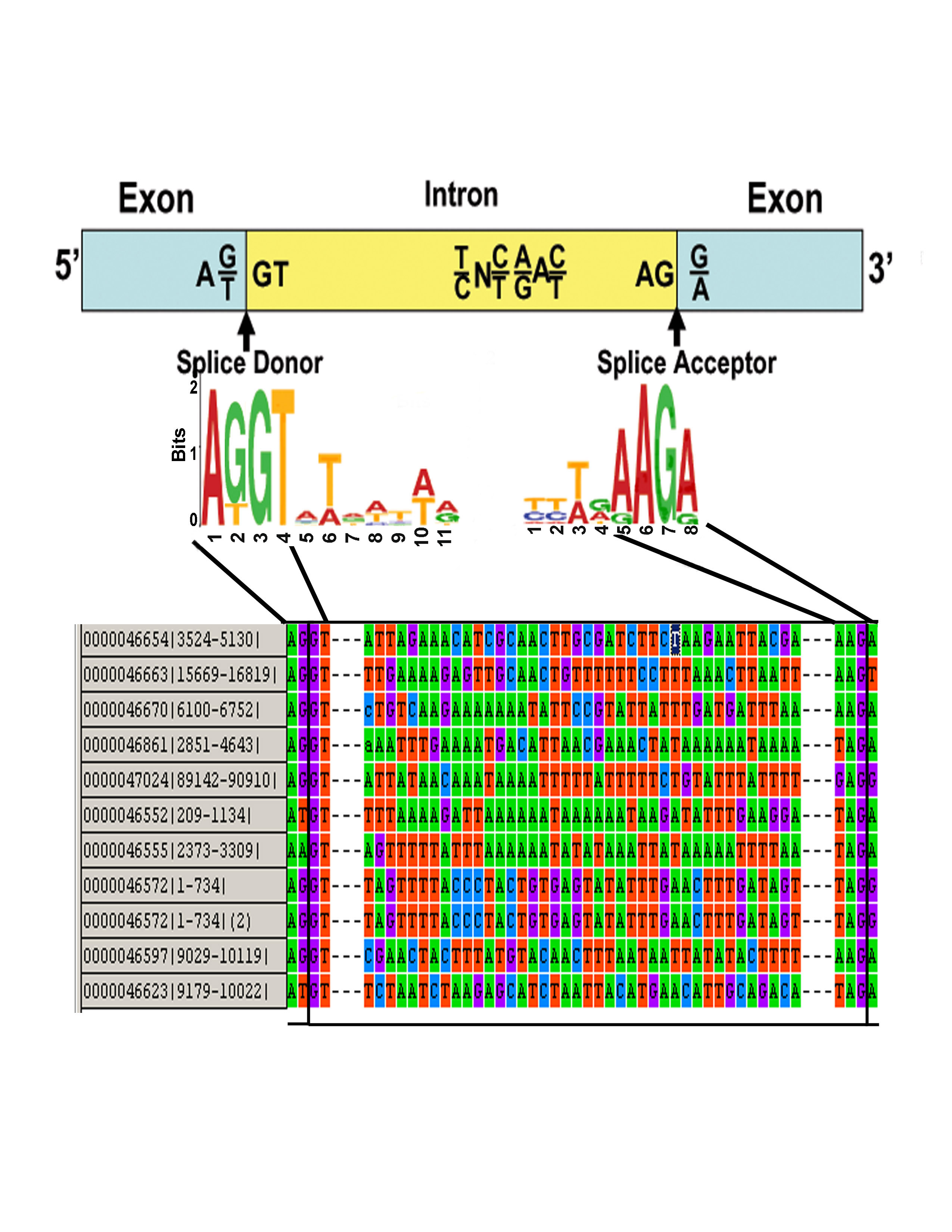

Supplement: Additional file 1: Figure S1 — Exon–intron junctions in most of the intron-containing protein-coding genes of N. apis. The consensus sequences “A(G/T)GT” at the donor site and “A(G/A)” at the acceptor site were indicated by arrows separately. [file 1471-2164-14-451-S1.tiff]

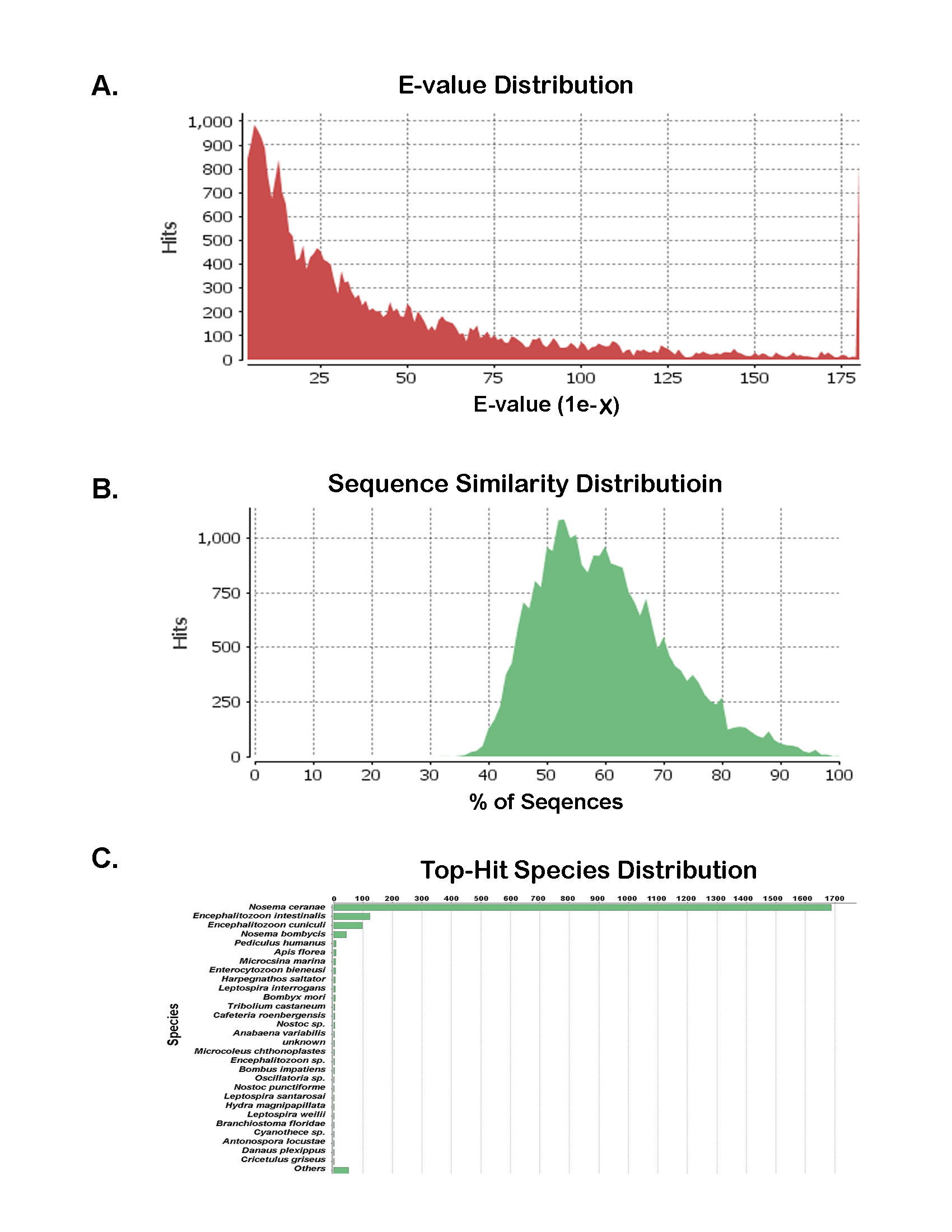

Supplement: Additional file 2: FigureS2 — Characteristics of homology searches of N. apis protein-coding genes against the non-redundant protein sequences (nr) at NCBI using Blastp. (a) E-value distribution of the top BLAST hit for each unique sequence with a cut-off E-value of 1.0e-3. The most sequences have an e-value between 1.0e-3 and 1.0e-25; (b) Similarity distribution of the top BLAST hit for each unique sequence. The sequence similarity of N. apis with database by Blast search ranges from 35% to approx 98% and peaks at 52%; and (c) Top-species distribution of the top BLAST hit for each unique sequence. The sequences of N. apis sequences showed the most significant similarity to the sequences of N. ceranae followed by sequences of E. intestinalis and E. cuniculi. [file 1471-2164-14-451-S2.tiff]

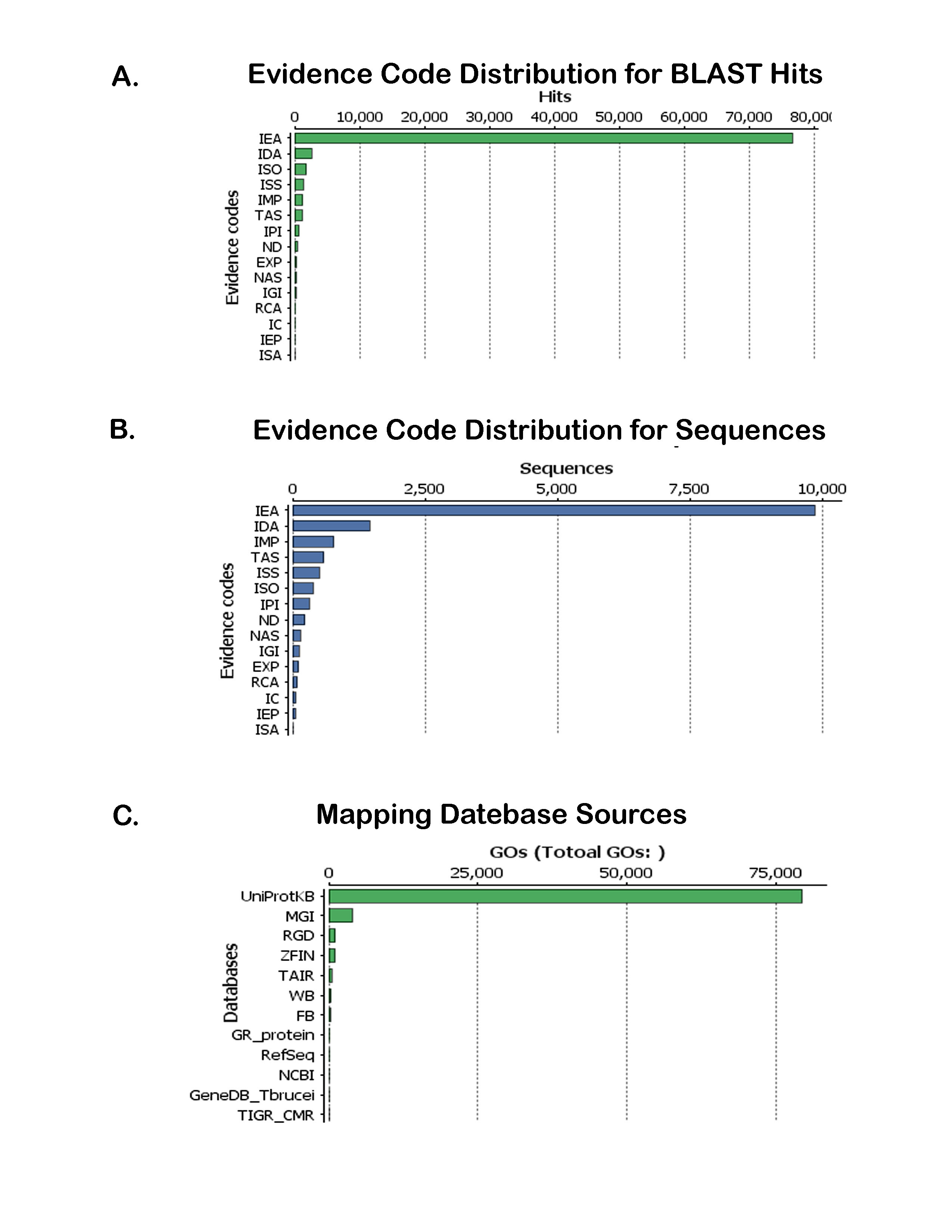

Supplement: Additional file 3: Figure S3 — Mapping of N. apis protein-coding genes to GO terms associated to Blastp hits. (a) Evidence code distribution for BLAST hits. The evidence code distribution for BLAST hits chart shows an overrepresentation of Inferred Electronic Annotation (IEA), followed by Inferred by Direct Assay (IDA); (b) Evidence code distribution for individual sequences. The highest evidence code for the individual sequences was through Inferred Electronic Annotation (IEA), second by Inferred by Direct Assay (IDA) and third by Inferred by Mutant Phenotype (IMP); (c) Mapping database sources. The majority of N. apis genes are obtained from the UniProt Knowledge Base (KB), a nonredundant protein database that includes PDS, UniProt, Swiss-Prot, TreMBL, and TAIR. [file 1471-2164-14-451-S3.tiff]

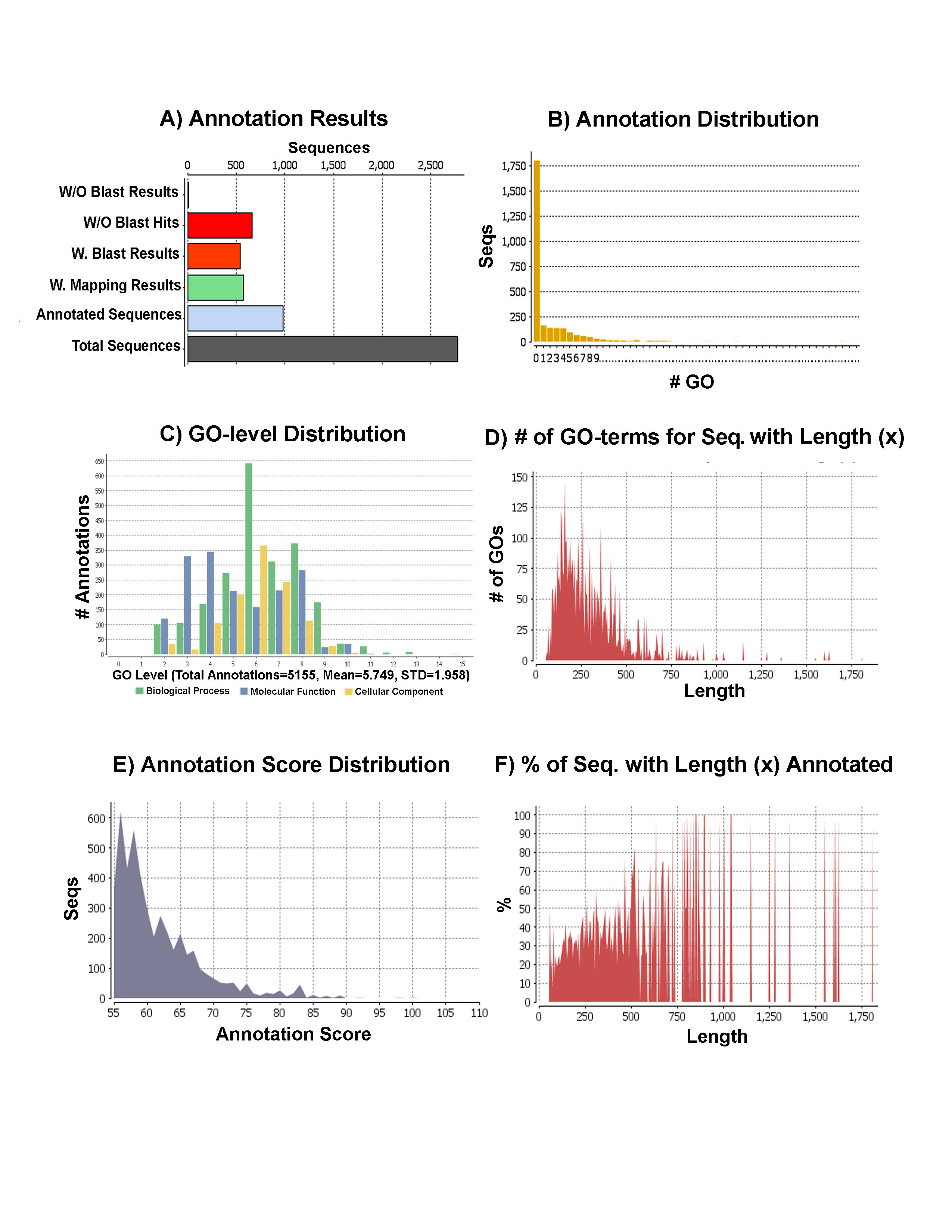

Supplement: Additional file 6: Figure S4 — Functional assignment terms to query sequences from the pool of GO terms gathered in the mapping step. (a) Annotation results. Of CDSs of N. aps with blast hit, 545 were assigned as conserved hypothetical proteins. The subsequent mapping assigned GO terms to 978 positive BLAST hit sequences. (b) Annotation distribution; Most sequences have between 1 and 6 GO terms annotated; (c) GO-level distribution. N. apis sequence GO terms representation for biological process (BP), molecular function (MF) and cellular component (CC) ontologies. The mean GO-level is 5.749 and 5155 annotations could be assigned; (d) Number of GO-terms for N. apis sequences with length (x). The length of most GO term annotated N. apis sequences were are in the 80–700 bp range; (e) Annotation score distribution; and (f) Percentage of N. apis sequences with length (x) annotated. [file 1471-2164-14-451-S6.tiff]
